# Microscale Engineering of n-Type Doping in Nanostructured Gallium Antimonide: AC Impedance Spectroscopy Insights on Grain Boundary Characterization and Strategies for Controlled Dopant Distribution

**DOI:** 10.3390/mi14091801

**Published:** 2023-09-21

**Authors:** Michael J. Hall, Daryoosh Vashaee

**Affiliations:** 1 Department of Materials Science and Engineering, NC State University, Raleigh, NC 27606, USA; mjhall3@ncsu.edu; 2 Department of Electrical and Computer Engineering, NC State University, Raleigh, NC 27606, USA

**Keywords:** GaSb (gallium antimonide), AC impedance spectroscopy, dopant distribution, microwave annealing, nanostructuring

## Abstract

This paper investigates the microscale engineering aspects of n-type doped GaSb to address the challenges associated with achieving high electrical conductivity and precise dopant distribution in this semiconductor material. AC impedance spectroscopy is employed as a reliable technique to characterize the microstructural and electrical properties of GaSb, providing valuable insights into the impact of grain boundaries on overall electrical performance. The uneven distribution of dopants, caused by diffusion, and the incomplete activation of introduced dopants pose significant obstacles in achieving consistent material properties. To overcome these challenges, a careful selection of alloying elements, such as bismuth, is explored to suppress the formation of native acceptor defects and modulate band structures, thereby influencing the doping and compensator formation processes. Additionally, the paper examines the effect of microwave annealing as a potential solution for enhancing dopant activation, minimizing diffusion, and reducing precipitate formation. Microwave annealing shows promise due to its rapid heating and shorter processing times, making it a viable alternative to traditional annealing methods. The study underscores the need for a stable grain boundary passivation strategy to achieve significant improvements in GaSb material performance. Simple grain size reduction strategies alone do not result in better thermoelectric performance, for example, and increasing the grain boundary area per unit volume exacerbates the issue of free carrier compensation. These findings highlight the complexity of achieving optimal doping in GaSb materials and the importance of innovative analytical techniques and controlled doping processes. The comprehensive exploration of n-type doped GaSb presented in this research provides valuable insights for future advancements in the synthesis and optimization of high-conductivity nanostructured n-type GaSb, with potential applications in thermoelectric devices and other electronic systems.

## 1. Introduction

Micro and nano-scale engineering has emerged as a crucial aspect in advancing the functionality of semiconductor materials. Through the control of material structure at these scales, it is possible to manipulate their electrical, thermal, and optical properties, vital for the miniaturization and enhancement of semiconductor devices [1,2,3]. Within this context, the field of semiconductors has made significant strides, innovatively employing nanostructuring to develop materials with unique electronic properties or low thermal conductivity [4,5,6,7]. This approach has expedited the development of advanced thermoelectric materials [8,9,10,11]. By pinpointing materials with a high power factor and applying processes that yield microstructural features under 100 nm, it is possible to curb thermal conductivity due to lattice vibrations [12]. The materials synthesized with grain sizes smaller than the typical mean free path of phonons but larger than that of electrons help reduce lattice contributions to thermal conductivity by acting as scattering centers. This meticulous control is particularly significant in the era of miniaturized devices, where material performance and device efficiency correlate with feature sizes and architectures at these scales [13,14,15]. Tremendous potential exists in significantly reducing lattice contributions to thermal conductivity while concurrently controlling the Fermi energy through strategic use of dopants, alloying, and nano-sized inclusions [16,17,18,19].

Gallium antimonide (GaSb), a prominent III-V compound semiconductor, exhibits properties that make it desirable for applications in infrared detectors, thermophotovoltaics, transistors, and thermoelectrics. In particular, the zincblende crystal structure of GaSb brings the energy level of the L-band close to the Γ band minima in the conduction band. As a result, n-type GaSb demonstrates transport in both bands. This band degeneracy is projected to influence thermoelectric properties by escalating the Seebeck coefficient (S), attributable to an increased effective curvature in the density of states at the conduction band edge, leading to an enhanced thermoelectric power factor. This enhancement can be leveraged by nanostructuring to reduce thermal conductivity and attain a large thermoelectric figure of merit (zT). The enhancement in thermoelectric performance through nanostructuring can be understood using standard methods. Grain boundaries are usually treated as scattering centers for phonons, which reduces the mean free path, thereby influencing the overall thermal conductivity of the material [17,20]. However, the process of n-type doping in GaSb presents unique challenges [21]. Uneven distribution of dopants, a consequence of diffusion, can lead to inconsistencies in the material’s properties. Further, not all introduced dopants are activated. For example, tellurium, a frequently used n-type dopant for GaSb, has a high activation energy, resulting in only a fraction of the introduced atoms being ionized and contributing to the n-type conductivity.

The problem of surface charge accumulation due to Fermi level pinning, common in III-V semiconductors, is amplified in n-type doping. This has notable effects on device performance, particularly in metal-oxide-semiconductor field-effect transistors (MOSFETs), where the surface plays a vital role. Moreover, the stability of the doping over time, especially at elevated temperatures, can be problematic due to the dopants’ diffusion or changes in their activation state. Oxidation, to which GaSb is particularly prone, can alter the surface properties, complicating the manufacturing process. The quality of the GaSb crystal is also a critical concern. Defects in the lattice can act as compensating centers, neutralizing some n-type dopants and reducing overall carrier concentration. 

One significant challenge is the migration of dopants into grain boundaries and the formation of larger precipitates, which can disrupt electrical uniformity. Understanding and mitigating these effects necessitates innovative analytical techniques and controlled doping processes. Success has been recorded with p-type GaSb, wherein the fusion of nanostructuring and Te compensator complex point defects significantly curtailed the lattice thermal conductivity of the material [22]. In this study, however, the goal is the investigation of n-type material, thus rendering this compensator counterproductive, as it would generate minority carriers, consequently reducing the magnitude of the Seebeck coefficient while increasing the electronic contribution to thermal conductivity due to bipolar conduction.

Under the solubility limit of ~4 × 10^18^ cm^−3^, Te serves as a shallow donor that substitutes for Sb [23,24]. Intriguingly, GaSb features two different native acceptors, believed to be Gallium vacancies (V_Ga_) and antisites (Ga_Sb_) [25]. These defects in meticulously melt-grown single crystals lead to a hole concentration of around 10^17^ cm^−3^ at 300 K [26], complicating the creation of high-quality n-type material. To create n-type GaSb with high electrical conductivity (σ), suppressing native p-type defects is essential, as these compensators act as traps and recombination centers, decreasing electron concentration and mobility [17].

A judicious selection of alloying elements, like bismuth, can help suppress the formation of native acceptor defects. Although no reported GaBi zincblende crystal exists [27], dilute bismides are frequently formed as alloys of III-V semiconductors. Bismuth substitutes for Sb in the zincblende lattice, preferentially filling V_Sb_ and leading to a reduction in residual acceptor concentration to ~10^16^ cm^−3^ in single crystalline samples [28,29,30]. Bismuth’s addition presents exciting prospects for tuning the bandgap of GaSb, potentially leading to the tunability of intrinsic carrier effects and optimizing the performance temperature range.

By studying the thermoelectric properties of the alloy, this research could shed light on the impact of Bi additions, which have been found to shift both the valence band by 9.6 meV and the conduction band −26.0 meV per at%, significantly affecting doping and compensator formation. These challenges require reliable techniques for their characterizations to enable stringent control over the manufacturing process and the development of surface passivation, oxidation protection, and crystal quality improvement. 

This paper will focus on the application of AC impedance spectroscopy to characterize the doping trends and the grain boundary effects in GaSb at microstructural scales. This method offers insightful information on the microstructural and electrical properties of GaSb, providing a clearer picture of how grain boundaries impact overall electrical performance. Moreover, in the realm of micromachines, where devices are being scaled down to micro and nano dimensions, understanding and optimizing the materials at these scales is indispensable. Additionally, we will study the effect of microwave annealing on activating the dopants. Given its rapid heating and shorter processing times, microwave annealing has shown promise in improving dopant activation, minimizing dopant diffusion, and reducing the formation of precipitates. This paper aims to explore these methods and their potential for overcoming the persistent challenges in GaSb n-type doping in the context of micro and nano-scale devices and systems.

## 2. Materials and Methods

The materials were synthesized through the careful measurement of elemental powders in precise stoichiometric ratios to achieve GaSb_1−x−y_Bi_x_Te_y_. This was achieved with varying x values of 0.0, 0.0025, 0.01, 0.025, and 0.05. All activities were conducted inside an Argon-filled glovebox. Gallium, which was a large chunk, was separately melted on a 40 °C hot plate and dispensed via a pipette. To ensure material purity, all powder precursors underwent heating under an active vacuum for a minimum of eight hours in the glovebox antechamber. The targeted Te doping concentration was set at 4 × 10^18^ cm^−3^, denoted by y ≈ 0.00023. A GaSb sample without any dopants was also synthesized to serve as a control sample.

Precise weighing of materials was achieved using a microbalance, guaranteeing an accuracy of 0.1 mg. The weighed materials were placed in quartz tubes and sealed under a vacuum of at least 10^−3^ torr. The accuracy of the balance allowed us to measure the components with required precisions of 4.1% for Te, 0.23% for Bi, 0.000098% for Sb, and 0.00017% for Ga for 8 g samples. To ensure homogeneity, samples were melted and agitated within the quartz. Rapid cooling was achieved by submerging the quartz ampoule in water under ambient conditions until the material completely solidified.

These solid samples were then annealed at 650 °C for 72 h. Post-annealing, the samples were sliced into suitable geometries for further characterization. X-ray diffraction analysis was conducted using a MiniFlex 600 (Rigaku, Tokyo, Japan) to determine crystallographic properties. A LSR-3 (Linseis, Selb, Germany) was employed to measure electrical conductivity (σ) and the Seebeck coefficient (S) as functions of temperature. To ascertain thermal diffusivity (α), a LFA 1000 (Linseis, Selb, Germany) was utilized for Laser Flash analysis. Using this data, combined with the literature c_p_ data and density (ρ) obtained by the Archimedes method, we calculated the total thermal conductivity (κ).

$$\kappa =c_{p}(T)\cdot \alpha (T)\cdot \rho (T)$$

The dimensionless figure of merit, zT, was calculated using the formula zT = (S^2^σT)/κ, in which S is the Seebeck coefficient, σ is the electrical conductivity, T is the absolute temperature, and κ is the total thermal conductivity.

The samples underwent further treatment where they were heated to 600 °C within a single-mode microwave cavity and subjected to pulsed radiation at a frequency of 2.45 GHz, a condition subsequently referred to as “Post-MW”. Following this microwave treatment, the characterization process was repeated on the treated samples.

## 3. Results and Discussion

**Figure 1:**
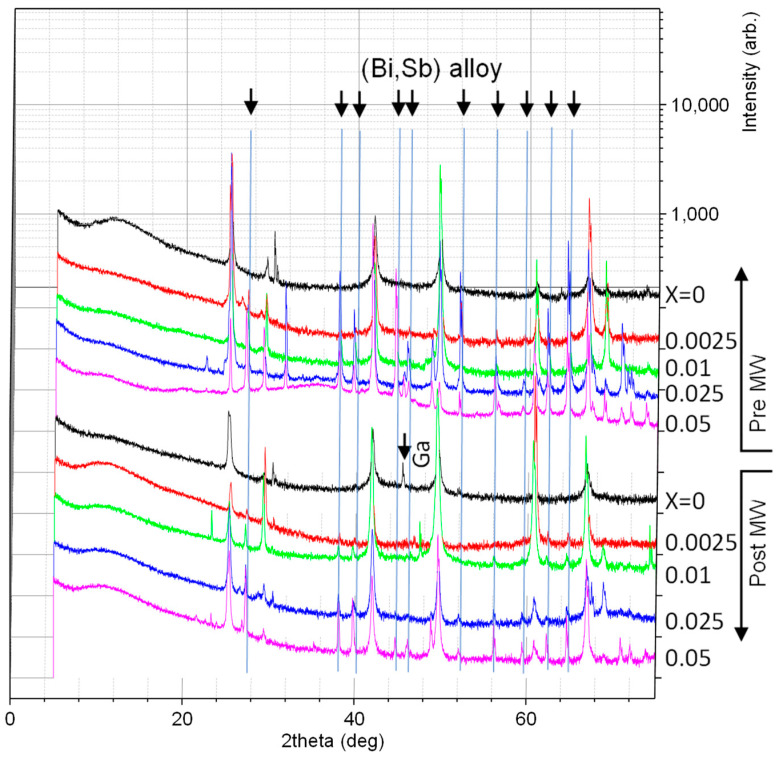
X-ray Diffraction data, with peak positions labeled for Ga and Bi_0.84_,Sb_0.16_ alloys as identified using PDXL2 software (version 2.9.1.0). Peaks not labeled correspond to those expected in GaSb.

**Figure 2:**
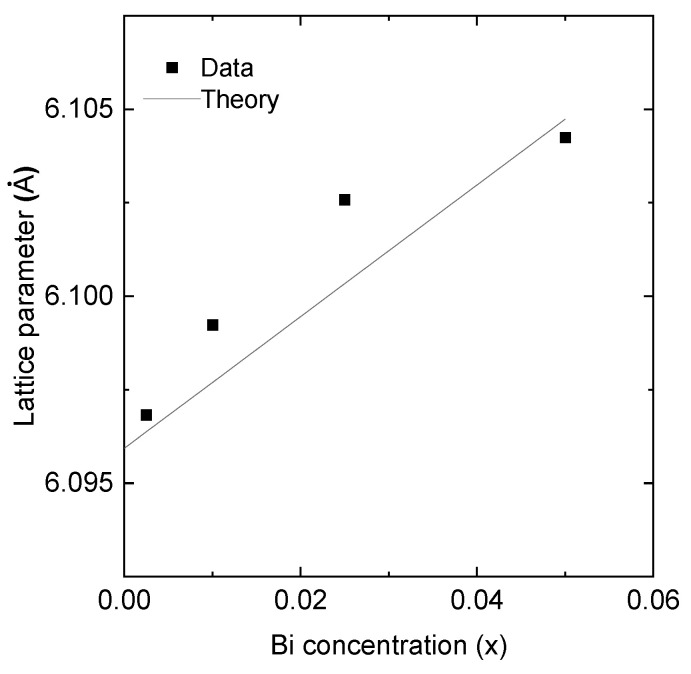
Lattice parameter determination based on the measured position of the (111) peak.

Insights from X-ray diffraction (XRD) data, as depicted in Figure 1, reveal an increase in lattice constant as a function of x, measured using the position of the (111) GaSb_1−x_Bi_x_ peak. This observation is in line with Vegard’s law, which predicts an increased lattice constant of 6.272 Å for the hypothetical GaBi zincblende crystal. This theoretical value is consistent with our experimental data, as illustrated in Figure 2.

Table 1 presents the estimated values for the target composition, density, and grain size of the synthesized samples. The grain size dynamics observed in the synthesized samples demonstrate a non-monotonic trend with respect to bismuth concentration. To elucidate this behavior, several factors were considered. Initially, as the bismuth concentration in the GaSb matrix begins to increase, the solubility limit of bismuth in GaSb approaches saturation. This results in the refinement of the grain structure in the early stages, largely attributed to the formation of smaller, Bi-induced nucleation sites that facilitate more rapid grain nucleation and hinder grain growth. However, upon further increasing the bismuth concentration, exceeding its solubility limit in GaSb might result in the formation of secondary Bi-rich phases. These phases can act as preferential sites for grain growth, leading to an increase in grain size. This phenomenon could explain the observed plateau and subsequent grain size increase. Furthermore, it is essential to acknowledge the impact of tellurium, which, although present in minor amounts, could interact synergistically with bismuth, affecting the overall grain dynamics. The combined effects of these factors, alongside potential thermal effects during processing, may result in the complex grain size evolution observed with varying bismuth concentrations.

The notable increase in grain size observed in the GaSb_0.94977_Bi_0.05_Te_0.00023_ sample was not entirely unexpected. This change can be attributed, at least in part, to the partial melting of the sample during microwave annealing. This observation suggests that the microwave treatment had a discernible impact on the microstructure of the material, which is an important factor in its thermoelectric performance.

Alterations in the relative intensities of diffraction peaks may signify texturing. This differs from the patterns typically observed in standard powder diffraction, where the dispersion of numerous grains often results in lower-angle peaks demonstrating higher intensity, due to the fundamental nature of diffraction scattering. Particularly in samples with Bi > 0.01, peaks at 2θ = 42(220) and 68(420) degrees are markedly taller than expected, suggesting the possibility of sample texturing during the initial melt-growth process.

The comparison of data from both pre- and post-microwave (MW) treatment indicates an enhanced degree of texturing after the heat treatment. This could be because the (220) faces, which have fewer bonds per unit area in zincblende crystals, might be preferred during the initial melt quench. The process would necessitate fewer bonds for continuous growth and lower solute diffusion requirements, facilitating rapid growth. Under these circumstances, further heat treatment is likely to accentuate the texturing effect as larger grains proliferate at the expense of smaller ones.

Figure 2 presents the determination of the lattice parameter based on the measured position of the (111) peak. In this figure, the observed curvature in the experimental data points towards a potentially intricate relationship between dopant concentration and the lattice constant. It is worth noting that our theoretical calculations utilized a linear approximation based on Vegard’s Law due to the absence of a system-specific bowing parameter in the existing literature. This discrepancy underscores the necessity for further investigation to derive a more accurate bowing parameter for this material system.

**Figure 3:**
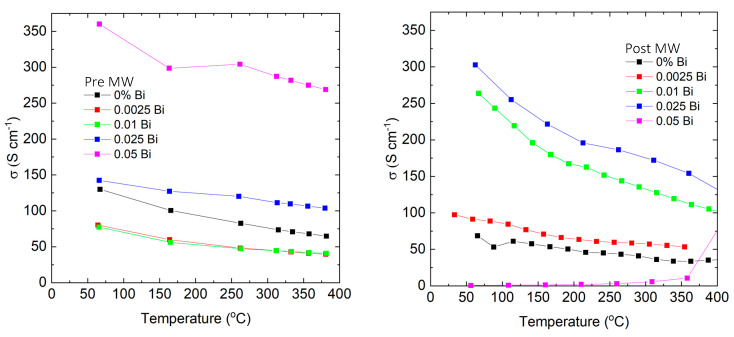
Electrical conductivity vs. temperature for samples quenched from melt before (**left**) and the same samples after (**right**) annealing in a single-mode microwave cavity.

As depicted in Figure 3, the electrical conductivity for the majority of the synthesized materials was less than the anticipated value. Previous studies on Zn-doped p-type GaSb have reported a Hall mobility between 100 and 160 cm^2^/Vs when the doping concentration is within 2–8 × 10^19^ cm^−3^ [31]. It is known that electron mobility in GaSb is substantially higher than hole mobility. Particularly in polycrystalline materials, electron mobility has been cited as being as high as 1200 cm^2^/Vs [32]. Drawing from these established figures, we projected our samples to exhibit a conductivity in the range of 600–1500 S/cm. However, our experiments yielded values that were markedly lower, spanning only 50–350 S/cm. This discrepancy can likely be attributed to an energy barrier, induced by both impurities and intrinsic defects in the grain boundaries [33]. Further examination of carrier transport properties will shed light on the correlation between the material’s resistance and the prevalence of competing transport phenomena.

**Figure 4:**
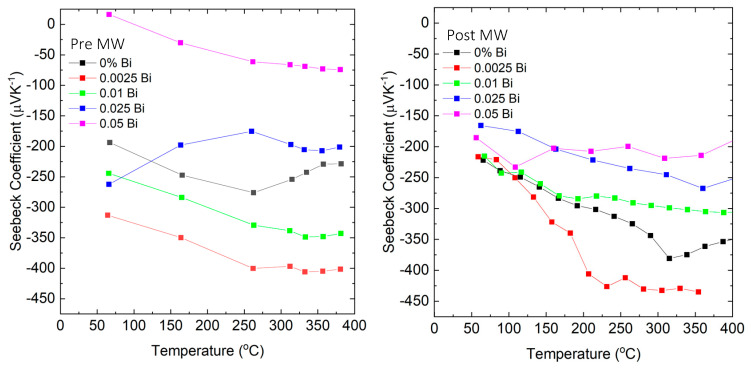
Seebeck coefficient vs. temperature for samples quenched from melt before (**left**) and the same samples after (**right**) annealing in a single-mode microwave cavity.

In Figure 4 the intricate dynamics of bismuth doping in GaSb are visually delineated, especially evident in low-concentration samples. Prior to microwave annealing, Bi introduces certain disturbances within the GaSb crystal lattice, likely due to localized defects or states that trap carriers. This is particularly significant when Bi compensates electron-donating interface defects. Under such circumstances, an increase in the absolute value of the Seebeck coefficient (Figure 4) and a subsequent decrease in electrical conductivity are discernible. Post microwave annealing, the narrative undergoes a transformation. The rapid, uniform heating characteristic of the microwave process seems to enable deeper diffusion of Bi into the material’s bulk. Within this domain, Bi has the propensity to compensate electron-accepting defects. This dynamic gives rise to an increased electron concentration, evident as a diminished absolute value of the Seebeck coefficient (Figure 4) coupled with a marked enhancement in electrical conductivity. 

Also, it is notable in Figure 3 that the conductivity of the sample doped with 0.05 Bi sees a significant reduction after microwave annealing. Preliminary insights into this behavior can be traced back to the structural integrity of the material. As detailed in the SEM analysis section, SEM examinations uncover multiple cracks in the GaSbBi_0.05_:Te post-annealing. These structural imperfections, likely stemming from localized melting events during the microwave process, are believed to be responsible for the sharp drop in conductivity.

Bismuth-added samples, prepared via melt quenching and annealing, present relatively consistent trends in their thermal and electrical transport properties. The sample with a Bismuth concentration of 0.05 achieved the highest electrical conductivity and simultaneously exhibited the lowest thermal conductivity. This observation can be elucidated using XRD data from this composition, which suggests the existence of a metallic phase, likely a Bi-Sb alloy. Given that elemental Bi possesses higher σ and lower κ than GaSb, the inclusions with 0.05 Bi might be sufficiently large or densely distributed to substantially impact the transport properties.

However, this interpretation leaves open the question of the fate of excess Ga in the material. While ambient temperature XRD might struggle to detect small quantities of Ga, particularly if it is in a liquid state, no evidence of liquid Ga was apparent when the samples were sliced for characterization. Interestingly, XRD peaks for Ga metal were observed in the 0% Bi material post microwave treatment, potentially due to field-induced separation, decrystallization, or growth of existing Ga inclusions.

When considering the resistivity contributions of the grain boundaries, multiple approaches can be adopted. One could treat them as a secondary phase with lower carrier concentration and mobility, exhibiting a weak temperature dependence. This approximation might suffice if the energy barriers are of the order of kT. However, for larger energy barriers, the current may be limited by thermionic emission, resulting in substantial resistivity reductions as temperature increases. For a degenerately doped semiconductor, the bulk conductivity typically decreases with a T^−3/2^ dependency due to increased scattering, assuming ionized impurity scattering as the dominant mechanism. The activation of transport across grain boundaries counteracts this temperature dependency, resulting in a significantly lower temperature dependence of resistivity.

A deviation from the temperature dependence of resistance provides further evidence that multiple carrier transport processes are at work in the synthesized material. The materials we studied, which demonstrated significant increases in electrical conductivity, showed an approximate T^−3/2^ dependency. This suggests that the grain boundaries’ influence on transport lessens after annealing in the single-mode microwave cavity (SMMC).

Moreover, the temperature dependency of conductivity shifted from a T^−1^ to a T^−1.35^ relationship. This shift suggests that the charge carrier transport is gradually approaching the typical behavior observed in degenerately doped semiconductors and is becoming less influenced by currents that flow solely due to thermionic emission.

In Figure 4, we present the Seebeck coefficient as a function of temperature for samples quenched from melt before microwave annealing (left) and for the same samples post microwave annealing in a single-mode microwave cavity (right). The distinct temperature-dependent behavior of the Seebeck coefficient for the GaSb_0.97477_Bi_0.025_Te_0.00023_ samples becomes evident in this figure. This trend can be attributed to the initial distribution and subsequent diffusion of Bi within the GaSb matrix. Before microwave annealing, the Bi atoms are not evenly distributed throughout the bulk material. This non-uniform distribution manifests as a pronounced difference in the Seebeck coefficient at lower temperatures. As temperature rises during the measurement, the gradual diffusion of Bi into the lattice contributes to the convergence of the Seebeck coefficient values. After microwave annealing, when the diffusion of bismuth atoms is believed to be complete within the GaSb lattice, the temperature-dependent behavior of the Seebeck coefficient in these samples aligns more closely with the expected trend observed in other samples.

All bismuth additions altered the relationship between the Seebeck coefficient (S) and temperature. Without bismuth, the thermopower displayed a negative temperature dependence above 300 °C, suggesting the occurrence of the bipolar effect due to thermally activated carriers (see Figure 4). This trend vanished in samples with added bismuth, hinting at a decrease in minority carrier concentration within the material across the studied temperature range.

Adding more bismuth led to an enhanced Seebeck coefficient, but this trend was inverted for compositions above 0.025 Bi. A contrasting trend appeared in the electrical conductivity, suggesting that the Fermi level is shifting towards the conduction band. This correlates with the trend in thermopower.

Increases in the Seebeck coefficient post microwave annealing at x = 0 and 0.05 can likely be linked to a decrease in carrier concentration as they coincide with a drop in σ. This suggests a reduced concentration of Te substituting into Sb sites, leading to fewer donor states post microwave. For instance, the Te could stop acting as a donor due to the formation of Ga_2_Te_3_ clusters with p-type conductivity.

**Figure 5:**
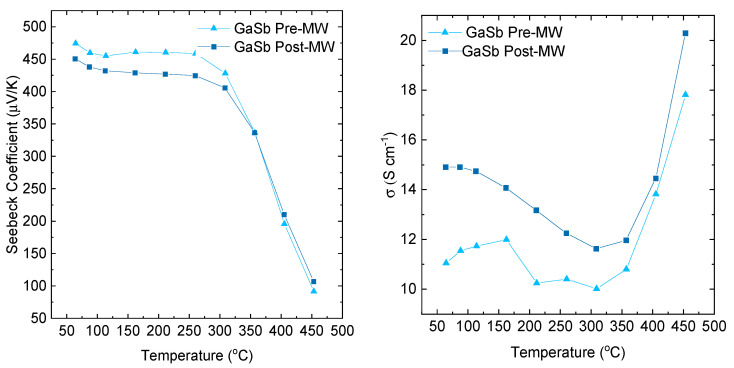
Comparison of Seebeck coefficient and electrical conductivity for a commercially-produced GaSb powder post densification via spark plasma sintering (SPS) and following subsequent microwave treatment.

Figure 5 illustrates that transport in undoped GaSb is dominated by native p-type defects, leading to a strong intrinsic carrier effect as temperature increases. This results in a strong temperature dependence on conductivity and the Seebeck coefficient.

## 4. AC Impedance Measurements

To ascertain the influence of grain boundaries on thermoelectric performance, precise electronic measurements can be used to detect potential energy barriers within the sample. In a degenerately doped semiconductor, thermionic field emission is likely the primary form of transport across a grain boundary, where compensating states establish an energy barrier that restricts the current able to cross. However, AC impedance spectroscopy could also be deployed to learn more about the grain boundary’s barrier height and shed light on potential improvements [34].

One less complex method for examining time-dependent conduction mechanisms than optoelectronic methods, particularly for bulk materials, is to measure the frequency dependence of resistivity. At low frequencies, the time taken for grain boundaries to deplete depends on the applied voltage, potentially resulting in voltage-dependent capacitance under the depletion approximation that ne and n_p_ equal zero. In this scenario, grain boundaries could function similarly to parallel plate capacitors.

There might be other notable features to observe, such as the voltage needed to activate thermionic emission and thermionic field emission. If the majority carrier concentration is known, the minority carrier concentration could be ascertained using the material’s dielectric constant and the effective width of the depletion region, determined by the current density.

**Figure 6:**
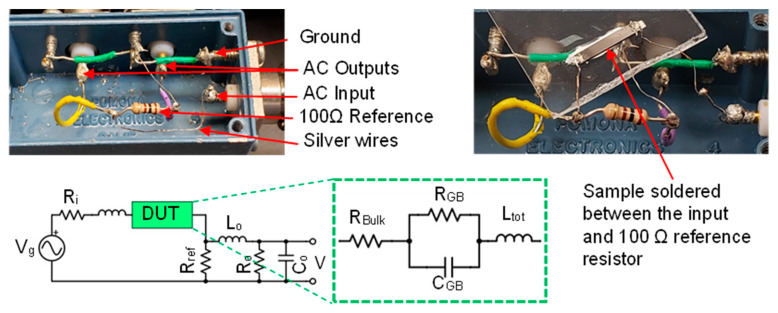
Circuit diagram of the AC impedance measurement setup and a model equivalent circuit representing a polycrystalline semiconductor sample with an energy barrier that permits leakage due to finite resistance in the charge-depleted regions at compensating-type grain boundaries. The inductor illustrates two phenomena: the inductance of the sample and the reduced capacitance of the energy barrier at high frequencies.

Frequency sweeps can be utilized to ascertain capacitance by simulating the response of an equivalent circuit composed of resistors and capacitors in series and parallel (refer to Figure 6). Should the modeled circuit vary based on the amplitude of excitation, these changes can be examined by integrating voltage and current dependencies into the circuit elements. If the equivalent circuit comprises a resistor in parallel with the other components, the voltage dependence of that resistor will be affected by leakage current from minority carriers, and the activation of tunneling, thermionic emission, or thermionic field emission. 

**Figure 7:**
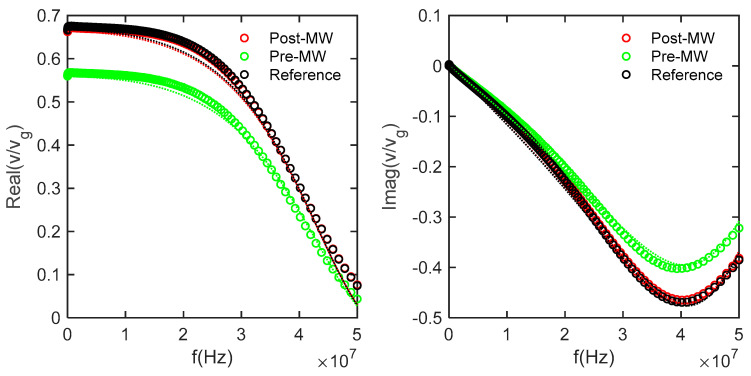
The reference curve is fitted by holding R_i_, R_o_, R_ref_, and V_g_ constant while varying C_o_, L_o_, and L_i_. Subsequently, preliminary values for R_bulk_, R_GB_, C_GB_, and L_tot_ were also successfully estimated.

**Figure 8:**
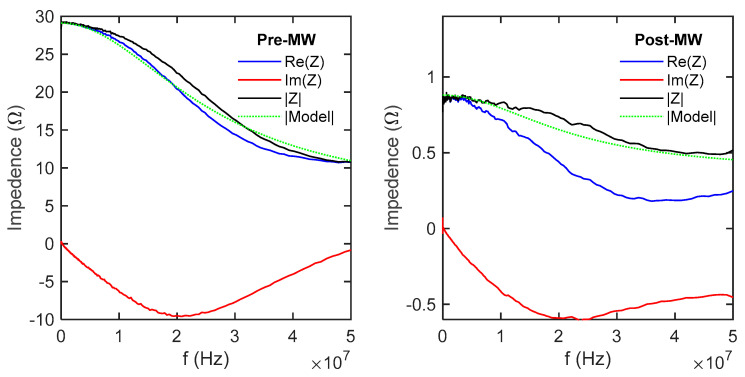
The impedance of the sample was determined by measuring the voltage across a reference circuit while sweeping the frequency. The known voltage on the reference circuit enabled the determination of the current in the sample, allowing for the calculation of the impedance.

The impedance measurements were conducted using a Zurich lock-in amplifier, covering a broad frequency range from 100 Hz to 50 MHz. The utilization of AC impedance with multiple excitation frequencies allowed for efficient scanning of different excitation voltages. To determine the appropriate parameter values for the model circuit, curve fitting techniques were applied, as demonstrated in Figure 7 and Figure 8. This fitting process accounted for the presence of parasitic inductances and capacitance originating from various sources, including the wires, reference resistor, plugs, and connectors in the signal generation and measurement circuits.

The studied samples were GaSb in both the as-quenched and MW-processed conditions. To facilitate resistance measurements while keeping the current within the lock-in amplifier’s 100 mA maximum limit, the samples were sliced using a wire saw and polished to a more convenient thickness. To minimize the influence of wire inductance, we employed short wires to transmit the signal into the input channel of the amplifier. Additionally, differential inputs were utilized, as this configuration enhances noise reduction through common mode rejection.

Despite high-frequency operation, the flow of current through the sample remains consistent with the principles of mass and energy conservation. It is important to consider the presence of some wire inductance in the system for high-frequency operations. However, by employing appropriate measures such as short wires and differential inputs, we aimed to minimize the impact of wire inductance on the measurements.

The analysis of both pre- and post-MW samples is detailed in Table 2. The shaded columns are values extracted by fitting the voltage across the reference resistor, while the unshaded columns represent fits obtained once the sample is incorporated into the circuit. It’s worth noting that, in the reference circuit, these elements are absent.

Figure 7 shows the voltage data v at the differential inputs of the lock-in amplifier, measured as a function of frequency and normalized to the generator voltage v_g_. The solid lines are the modeled voltage that would be measured based on the circuit elements, which were iteratively fit to the reference data measured across a 100 Ω resistor. Figure 8 takes the next step where the modeled resistance of the whole system was used to determine the current flowing through the samples to calculate the real, imaginary, reactive, and total impedance.

The frequency-dependent impedance of thermoelectric materials can be partially explained by the interaction of heat transfer mechanisms, specifically the Peltier effect and thermal conduction at the material’s thermal contacts, which occurs at a different rate than the flow of electrical current. In this study, thin thermoelectric samples are securely mounted on glass slides using Zircar paste, providing mechanical stability for soldering wires to the ends. Consequently, at low temperatures, the majority of heat transfer in and out of the sample primarily occurs through the relatively high-mass glass slide and the Zircar paste. In an idealized model, the thermoelectric elements can be treated as Warburg elements [35], characterized by a phase angle resulting from the disparity in transport properties between heat current (Ω_Q_) and electrical current:

$$\begin{array}{c} \Omega_{Q}={\frac{S^{2}TL}{\kappa A_{e}}\left(\frac{A_{t}}{t^{2}}+\frac{j\omega }{\omega_{c}}\right)}^{-0.5}tanh{\left(\frac{A_{t}}{t^{2}}+\frac{j\omega }{\omega_{c}}\right)}^{0.5}\\ \omega_{c}=\alpha t^{2} \end{array}$$

**Figure 9:**
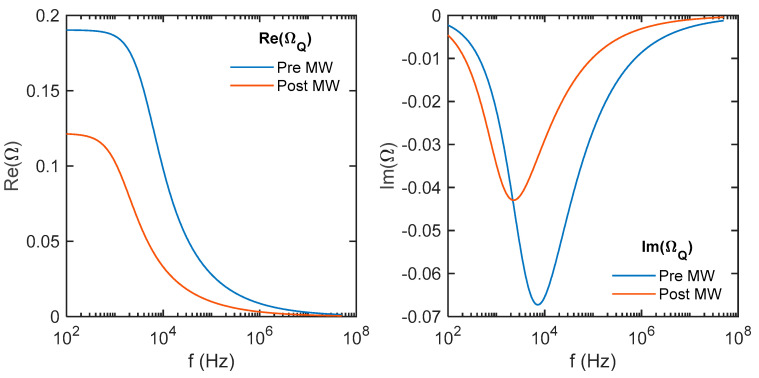
The calculated impedance resulting from the heat transferred within the sample through the Peltier effect, followed by its conduction into the glass slide to which the samples are mounted.

Here, S is the Seebeck coefficient, T is temperature, L is sample length, κ is thermal conductivity, A_e_ and A_t_ are the cross-sectional areas of electrical and thermal conduction, t is thickness, α is the thermal diffusivity of the material, j is $\sqrt{-1}$, ω_c_ is the characteristic thermal frequency, and ω is the angular frequency of a given measurement. In Figure 9, the ideal impedance resulting from the heat currents (Ω_Q_) is calculated based on various measured values and the dimensions of the sample. The calculated values, although relatively low, do not exert a significant influence on the pre-MW state. However, they may offer an explanation as to why the simple model failed to accurately represent the post-MW sample, particularly in terms of the reactive part of the impedance. This discrepancy arises because the measured value of the reactive impedance could not be reached in the model, primarily due to the occurrence of the maximum imaginary impedance (Im(Z)_max_) of R‖C when the current flowing through both elements is equal. Specifically, Im(Z)_max_ is equal to R/2.

This effect, stemming from the heat current, results in relatively larger errors for thicker samples with lower resistance, while materials where the resistance is primarily influenced by grain boundaries are minimally affected. Therefore, it becomes apparent that the impact of this heat current-induced effect introduces proportionally greater deviations in thicker and less resistive samples, while materials characterized by significant resistance contributions from grain boundaries experience minimal consequences.

The inductance of a rectangular conductor (or busbar) is calculated at low frequencies using the equation [36,37]:

$$L_{busbar}=\frac{\mu_{0}}{2\pi }l\left\lfloor ln\left(\left(\frac{2l}{w+t}\right)+\frac{1}{2}+\frac{2}{9}\left(\frac{w+t}{l}\right)\right)\right\rfloor$$

Here, µ_0_ is the permeability of free space, l is the length of the conductor, w is the width, and t is the thickness. The definition of low frequency in this context is based on the condition where the skin depth is greater than half the thickness of the material (t/2). At high frequencies, the inductance decreases due to the reduced partial inductance of the inner region of the busbar, as the current becomes confined towards the surface.

To calculate the skin depth, we utilize the measured resistivity (ρ) at the frequency f_0_ of 50 MHz and a magnetic permeability (µ_r_) of ~1, obtained from a magnetic susceptibility of −38.4(10^−6^) cm^3^/mol. The skin depth is determined using the following formula:

$$Skin\;Depth\;(\delta )=\sqrt{\frac{\rho }{\pi f_{0}\mu_{r}\mu_{0}}}$$

By applying this calculation, we determined the skin depth for the samples under investigation to be 1.2 mm in the pre-MW state and 0.39 mm in the post-MW state. Since these values are greater than half the sample thickness (approximately 0.2 mm), it can be inferred that surface currents are not expected to have a dominant effect on the inductance. Consequently, the calculated inductance values for the busbar samples are 11 nH for the pre-MW sample and 11.9 nH for the post-MW sample, respectively.

**Figure 10:**
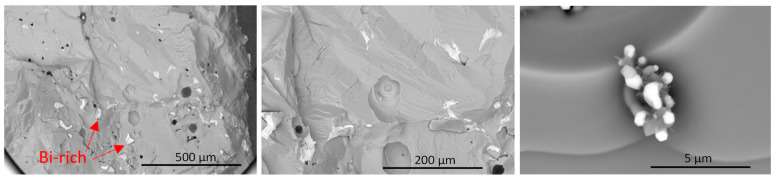
Backscattered electron images of GaSbBi_0.0025_:Te fracture surface, revealing bright regions indicating the presence of Bi-rich areas due to atomic number contrast from scattering.

A comparison of the low-magnification fractography images reveals notable distinctions between the samples. In Figure 10, the presence of smooth and curvy shapes in GaSbBi_0.0025_:Te indicates brittle crack propagation within the crack plane, originating from small voids or defects near the crack tip.

**Figure 11:**
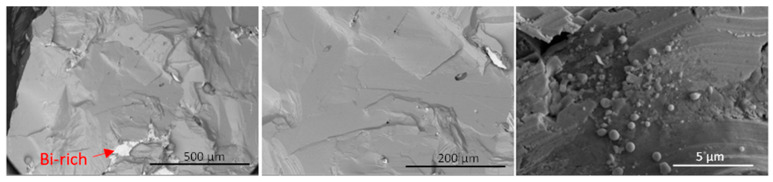
Backscattered electron images of GaSbBi0.05:Te fracture surface, exhibiting numerous cracks crossing the fracture plane. These cracks potentially contribute to the remarkably low electrical conductivity observed in this material after microwave treatment. Localized melting events can lead to substantial residual strains due to the volume expansion upon resolidification, which may explain the observed conductivity reduction.

Conversely, Figure 11 displays straight cracks with different orientations, lacking smooth curving structures. This suggests that some of the cracks observed on the surface existed prior to fracture were not a result of the brittle fracture crack opening force. 

**Figure 12:**
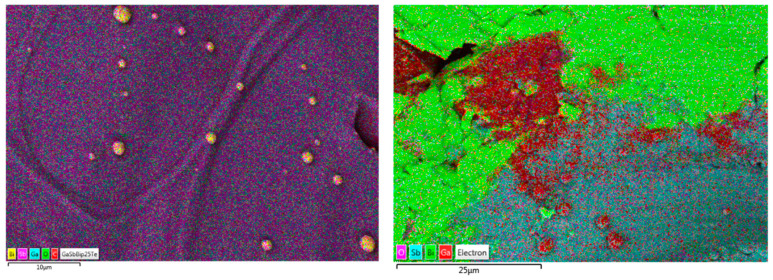
EDS layered maps revealing the presence of Bi-rich regions in the GaSbBi_0.0025_ (**left**) and GaSbBi_0.05_ (**right**) samples. These Bi-rich regions correspond to the bright particles observed in the backscattered SEM images.

Figure 12 shows the variation in phase composition, exhibiting a Bi-rich phase with different morphologies depending on the Bi concentration.

The materials under investigation possess a polycrystalline nature, which introduces a range of grain boundaries exhibiting different behaviors. This variability arises from the random tilting angles of the grains, leading to variations in defect density throughout the material.

**Figure 13:**
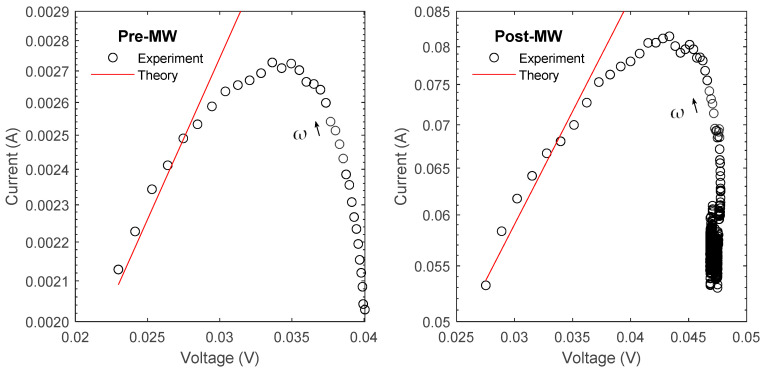
The Richardson equation was adapted to incorporate the applied voltage and fitted to the experimental data by adjusting the energy barrier height relative to the bulk Fermi level.

The I-V characteristics were determined by utilizing the measured impedance and the applied voltage across the sample at each frequency during the scan. The variation in the applied voltage is inherent due to the utilization of a fixed amplitude signal in a circuit with frequency-dependent impedance, notably the capacitance and inductance in the analyzer circuit. To obtain a comprehensive understanding, the fitting parameters for both samples are compared in Figure 13, and these parameters were employed to fit the red lines illustrated in Figure 13.

At high frequencies within the measured range, the I-V characteristics were successfully fitted using a voltage-dependent Richardson equation:

$$j=A^{*}T^{2}e^{-\frac{e\phi_{b}+E_{f}-ev_{s}}{k_{b}T}}$$

Here, A* represents the reported value of the thermionic emission constant [38], allowing for an approximate calculation of the energy barrier height relative to the Fermi level. This computation has been detailed in Table 3, which captures the calculated energy barrier heights from this analysis. In this equation, j denotes the measured current density resulting from thermionic emission, T represents the ambient temperature (300 K), ν_s_ represents the voltage applied to the sample, and eϕ_b_ + E_f_ corresponds to the height of the energy barrier relative to the Fermi energy, where the Fermi energy is considered as 0 for conduction electrons. Additionally, e and k_b_ represent the charge of an electron and Boltzmann’s constant, respectively.

According to this analysis, and as illustrated in Table 3, the energy barrier relative to the Fermi energy for GaSb_0.9877_Bi_0.01_Te_0.0023_ decreased from 0.3884 V to 0.3223 V after microwave (MW) annealing. It is important to note that in this model, all grains and grain boundaries were combined into series-limit elements. Therefore, these values are more representative of the cumulative total effects of multiple barriers within the material, rather than a single barrier.

The inconsistency between the low-frequency measurements and the thermionic emission model, observed in Figure 13, can be attributed to the competition among different transport mechanisms involved in steady-state current flow. Our model suggests that at low frequencies, current flow is primarily associated with the “leaky” or resistive part of the grain boundary. In contrast, at high frequencies, the current tends to flow through the capacitor or energy barrier rather than bypassing it. This frequency-dependent charge transport mechanism aligns with the trend observed in F, where the total resistance decreases as frequency increases and converges to a new lower value with less impact from the grain boundary capacitance.

At low frequencies, the buildup of space charge near the grain boundaries leads to the movement of the depletion region edges. The charges that move in and out of this region do not necessarily contribute to conduction, similar to the process of using an electric field to control the position of the p-region in an n-p-n junction. In thermoelectric devices and conductivity measurements conducted at relatively low voltages, the p-region only contributes to charge transport through carrier diffusion in the opposite direction of the induced electron current. Consequently, the low-frequency leakage current is limited by the concentration and mobility of holes in the grain boundary.

In contrast, at higher frequencies, the edges of the depletion region exhibit a reduced response to the electric field, resulting in lower energy losses. The current through the grain boundaries follows the thermionic emission current, where carrier mobility becomes less relevant as the transported carriers possess sufficient energy to be emitted past the grain boundary without scattering.

This explanation reasonably accounts for the differences observed between the samples in both the low- and high-frequency regimes. In the pre-MW condition, the lower capacitance and higher barrier height lead to higher resistance at low frequencies, primarily due to lower carrier mobility through wider depleted regions. At higher frequencies, the higher barrier height results in reduced thermionic emission. Assuming that the Fermi energy is partially pinned at the grain boundaries due to trap states, changes in the electron concentration in the bulk can influence both the width of the depletion region associated with the grain boundaries and the barrier height. This alignment is consistent with calculations showing lower bulk resistance (R_bulk_) and higher grain boundary capacitance (C_GB_) in the post-MW state, reflecting the higher electron concentration.

Grain size estimation was performed using the Halder–Webber peak analysis method implemented in the PDXL2 software package (version 2.9.1.0), taking into account the internal broadening standard. The results revealed significant increases in thermal conductivity across most of the materials, with the sample having an x value of 0.05 exhibiting the greatest enhancement. Notably, this sample also exhibited the largest relative change in crystal size, further highlighting the correlation between grain size and the observed increase in thermal conductivity.

**Figure 14:**
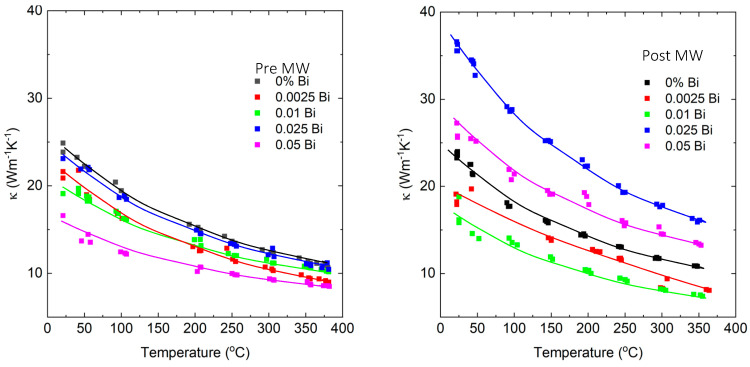
Thermal conductivity vs. temperature for samples quenched from melt (**left**) and after microwave annealing (**right**).

Upon quenching the samples from a melt, a general trend is observed: increasing Bi concentration leads to a decrease in thermal conductivity. However, this trend undergoes a significant change after microwave annealing. For materials with concentrations ranging from 0 to 0.01 B, the thermal conductivity decreases. In contrast, materials with higher Bi concentrations exhibit notable increases in thermal conductivity, as depicted in Figure 14.

X-ray diffraction (XRD) analysis was conducted before and after microwave annealing to examine the microstructure and composition of the annealed material in the solid-state microwave cavity (SMMC). The XRD results suggest that the increase in thermal conductivity for these samples can be attributed to a combination of grain growth, which is more pronounced in certain samples, and texturing. The laser flash measurement, which employs thin samples to minimize in-plane heat losses, further exacerbates the effect of texturing.

**Figure 15:**
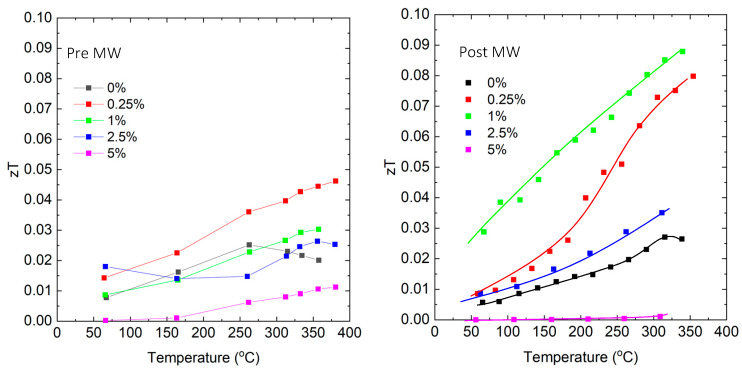
Dimensionless figure of merit (zT) vs. temperature for samples quenched from melt (**left**) and after microwave annealing (**right**). Solid lines are guides for the eyes.

Figure 15 demonstrates that the most substantial changes in the figure of merit (zT) after microwave annealing occurred in GaSb_0.9877_Bi_0.01_Te_0.0023_. Surprisingly, this particular sample underwent the least amount of grain growth yet exhibited significant alterations in the temperature dependence of electrical conductivity. This observation provides evidence that grain boundary limitations on transport were reduced in this sample.

## 5. Conclusions

In conclusion, this paper investigated the n-type doped GaSb, focusing on the challenges of achieving high electrical conductivity and controlled dopant distribution. We employed AC impedance spectroscopy as a central technique for characterizing the material’s electrical properties, which shed light on the influence of grain boundaries on overall conductivity. Our study revealed that the migration of dopants into grain boundaries and the formation of large precipitates significantly hinder electrical uniformity. Moreover, we observed that solely reducing grain size was insufficient in improving the thermoelectric properties, while an increase in grain boundary area compounded the issue through free carrier compensation. These observations highlighted the necessity for a more stable and effective grain boundary passivation strategy. Additionally, we explored the incorporation of alloying elements, notably bismuth, as a potential approach to counteracting native acceptor defects. This approach demonstrated promise in modulating band structures and consequently impacting the doping process. Another aspect of this research was the examination of microwave annealing as an alternative to traditional annealing methods. With its rapid heating and shorter processing times, microwave annealing exhibited potential in enhancing dopant activation, limiting diffusion, and reducing precipitate formation. This indicates that microwave annealing could be a more efficient and reliable method for the dopant activation process in GaSb materials. In sum, this research examines the multifaceted challenges of n-type doping in GaSb and explores techniques for controlled processing. The insights garnered contribute to the broader understanding of strategies to enhance electrical conductivity in GaSb-based devices. These findings serve as a stepping stone for continued development and refinement of nanostructured n-type GaSb materials.

## Figures and Tables

**Table 1:** Estimated values for target composition, density, and grain size of the synthesized samples.

Target Composition	Density (g/cm^3^)	Pre-MW Grain Size (nm)	Post-MW Grain Size (nm)
GaSb	5.51	51	---
GaSb_0.99977_Te_0.00023_	5.59	165	233
GaSb_0.99727_Bi_0.0025_Te_0.00023_	5.58	154	174
GaSb_0.98977_Bi_0.01_Te_0.00023_	5.60	95	75
GaSb_0.97477_Bi_0.025_Te_0.00023_	5.55	95	150
GaSb_0.94977_Bi_0.05_Te_0.00023_	5.61	272	546

**Table 2:** R_bulk_ R_GB_ C_GB_ and L_tot_ are the experimental data fits while the shaded columns were determined from the reference data curve and known values where applicable.

	R_bulk_	R_GB_	C_GB_	L_tot_	R_ref_	R_i_	V_g_	L_i_	R_o_	C_o_	L_o_
Pre-MW sample	5.9 Ω	23.2 Ω	0.26 nF	33 nH	100 Ω	50 Ω	100 mV	85 nH	1 MΩ	65 pF	90 nH
Post-MW sample	0.37 Ω	0.51 Ω	14 nF	30 pH	100 Ω	50 Ω	100 mV	85 nH	1 MΩ	65 pF	90 nH

**Table 3:** Calculated energy barrier from the line fits to the log-linear data in Figure 13. The Richardson equation was adapted to incorporate the applied voltage and fitted to the experimental data by adjusting the energy barrier height relative to the bulk Fermi level.

	Pre MW	Post MW
A* (A/cm^2^K^2^)	5.16 [38]	5.16
$e\phi_{b}+E_{f}$ (eV)	0.39	0.32
